# H3K36 methyltransferase NSD1 regulates chondrocyte differentiation for skeletal development and fracture repair

**DOI:** 10.1038/s41413-021-00148-y

**Published:** 2021-06-07

**Authors:** Rui Shao, Zhong Zhang, Zhan Xu, Huiling Ouyang, Lijun Wang, Hongwei Ouyang, Matthew Greenblatt, Xi Chen, Weiguo Zou

**Affiliations:** 1 Shanghai Institute of Microsurgery on Extremities, and Department of Orthopedic Surgery, Shanghai Jiao Tong University Affiliated Sixth People’s Hospital, Shanghai, China; 2 State Key Laboratory of Cell Biology, Shanghai Institute of Biochemistry and Cell Biology, CAS Center for Excellence in Molecular Cell Sciences, Chinese Academy of Sciences, University of Chinese Academy of Sciences, Shanghai, China; 3 Dr. Li Dak Sum & Yip Yio Chin Center for Stem Cell and Regenerative Medicine, Zhejiang University-University of Edinburgh Institute, Zhejiang University, Hangzhou, Zhejiang China; 4 China Orthopedic Regenerative Medicine Group, Hangzhou, Zhejiang China; 5 Department of Pathology and Laboratory Medicine, Weill Cornell Medicine, New York, NY USA; 6 Department of Molecular and Cellular Biology, Lester and Sue Smith Breast Center, Dan L. Duncan Cancer Center, Baylor College of Medicine, Houston, TX USA

**Keywords:** Bone, Pathogenesis

## Abstract

Chondrocyte differentiation is a critical process for endochondral ossification, which is responsible for long bone development and fracture repair. Considerable progress has been made in understanding the transcriptional control of chondrocyte differentiation; however, epigenetic regulation of chondrocyte differentiation remains to be further studied. NSD1 is a H3K36 (histone H3 at lysine 36) methyltransferase. Here, we showed that mice with *Nsd1* deficiency in Prx1^+^ mesenchymal progenitors but not in Col2^+^ chondrocytes showed impaired skeletal growth and fracture healing accompanied by decreased chondrogenic differentiation. Via combined RNA sequencing (RNA-seq) and chromatin immunoprecipitation sequencing (ChIP-seq) analysis, we identified sex determining region Y box 9 (*Sox9*), the key transcription factor of chondrogenic differentiation, as a functional target gene of NSD1. Mechanistically, NSD1 regulates *Sox9* expression by modulating H3K36me1 and H3K36me2 levels in the *Sox9* promoter region, constituting a novel epigenetic regulatory mechanism of chondrogenesis. Moreover, we found that NSD1 can directly activate the expression of hypoxia-inducible factor 1α (HIF1α), which plays a vital role in chondrogenic differentiation through its regulation of *Sox9* expression. Collectively, the results of our study reveal crucial roles of NSD1 in regulating chondrogenic differentiation, skeletal growth, and fracture repair and expand our understanding of the function of epigenetic regulation in chondrogenesis and skeletal biology.

## Introduction

Human stature depends mainly on the growth of long bones. Chondrocyte differentiation in the growth plate is a major factor for bone growth and is involved in endochondral ossification, the process that vertebrates use mainly to form the skeleton.^1^ Endochondral ossification begins with mesenchymal progenitor condensation to form chondroprogenitor cells, and chondrogenic differentiation, proliferation, and hypertrophy follow. Finally, blood vessels, osteoblasts, and osteoclasts invade the hypertrophic zone to generate cancellous bone.^2^ For chondrogenic differentiation, sex determining region Y box 9 (*Sox9*) is the key regulator and activates the chondrocyte-specific enhancer of *Col2* (collagen II). Mice with *Sox9* haploinsufficiency present defective primordial cartilage and abnormal skeletal mineralization.^3^ The transcriptional regulation of *Sox9* expression has been extensively reported; for example, hypoxia-inducible factor 1α (HIF1α) directly binds to the promoter of *Sox9* and activates *Sox9* expression, affecting early skeletogenesis.^4–6^

Accumulating evidence indicates that epigenetic modifications play important roles during chondrogenic differentiation and longitudinal bone growth. The histone demethylase PHF2 can stimulate chondrogenesis by binding to the promoter region of chondrocyte-related genes and removing H3K9me2 from these genes.^7^ Both KDM4B, an H3K9me3 demethylase, and KDM6B, an H3K27me2/3 demethylase, play crucial roles in chondrogenesis.^8,9^ Mutations in *KMT2D* or *KDM6A* are causes of Kabuki syndrome, characterized by mild-to-moderate intellectual disability, typical facial features, and short stature.^10,11^ Combined loss of the H3K27 methyltransferases EZH1 and EZH2 in mice severely impairs skeletal growth by affecting chondrogenesis in the growth plate and chondrocyte proliferation and hypertrophy.^12^ Beyond these processes related to H3K9 and H3K27 regulation, epigenetic regulation of chondrocyte differentiation and skeletal development remains to be further studied.

Nuclear receptor binding SET domain-containing protein 1 (NSD1), encoded by the *Nsd1* gene, catalyzes the mono- and dimethylation of histone H3 at lysine 36 (H3K36).^13^ In the clinic, deletion or mutation of the *NSD1* gene are the major causes of Sotos syndrome (cerebral gigantism),^14,15^ a genetic disorder with increased bone growth during infancy and childhood and normal height after puberty,^16^ strongly indicating that NSD1 is associated with bone growth. Histone H3 lysine 36 to methionine (H3K36M) mutation leads to decreased H3K36 methylation levels, which impairs mesenchymal progenitor differentiation and leads to undifferentiated sarcoma in mice and chondroblastoma in clinical patients,^17,18^ suggesting that H3K36 methylation plays important roles during chondrogenic differentiation and cartilage development; however, the exact enzyme that plays the key role has not yet been identified.

To explore the role of NSD1 and H3K36 methylation in chondrogenic differentiation and skeletal growth, we conditionally deleted *Nsd1* in mesenchymal progenitors by mating *Nsd1*^*f/f*^ mice with *Prx1-Cre* mice and in chondrocytes by mating *Nsd1*^*f/f*^ mice with *Col2*-*Cre* mice. Strikingly, we found that deletion of *Nsd1* in Prx1^+^ mesenchymal progenitors but not in Col2^+^ chondrocytes led to impaired skeletal growth and fracture healing in mice. Mechanistically, NSD1 regulated chondrogenic differentiation by controlling the expression of *Sox9* through direct regulation by modulating the occupancy of H3K36me1 and H3K36me2 on the *Sox9* promoter and indirect regulation by binding to the *Hif1α* promoter. These findings identified NSD1 as a novel regulator of chondrogenic differentiation, skeletal growth, and fracture healing, providing new insights into epigenetic regulation of chondrogenic differentiation and bone growth.

## Results

### Mice with *Nsd1* knockout in mesenchymal progenitors showed impaired cartilage development

**Fig. 1:**
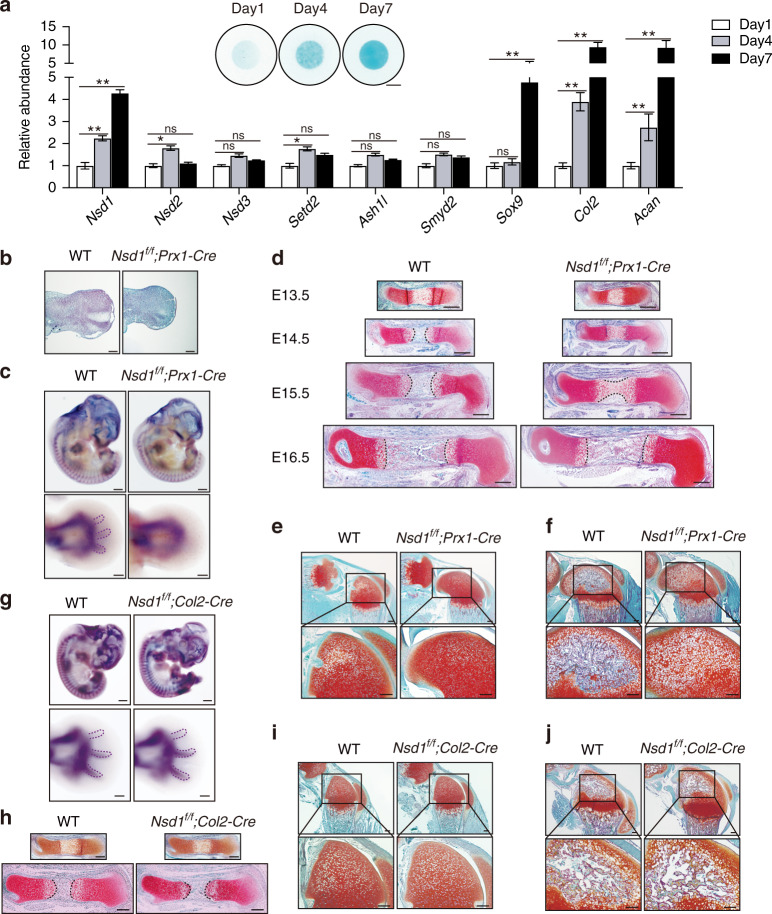
Mice with *Nsd1* knockout in mesenchymal progenitors showed impaired cartilage development. **a** mRNA levels of H3K36 methyltransferases and chondrocyte differentiation marker genes were determined by qRT-PCR in micromasses at different differentiation time points. The values are presented as the means ± SEMs, *n* = 4. *P < 0.05, **P < 0.01, ns means not significant. The inset shows Alcian blue staining results of micromasses cultured for 1, 4, and 7 days with chondroprogenitor cells. Scale bar = 2 mm. **b** SO staining results of E11.5 limb buds. Scale bar = 100 μm. **c** Whole-mount in situ hybridization (WISH) results for *Col2* in E12.5 embryos (top) and sections of forelimbs (bottom). The dashed purple lines show the digits already present. Scale bar (top) = 500 μm, scale bar (bottom) = 200 μm. **d** SO staining results of E13.5 (first line), E14.5 (second line), E15.5 (third line), and E16.5 (fourth line) femur sections from WT and *Nsd1*^*f/f*^*;Prx1-Cre* mice. The dashed black lines show the borders between hypertrophic chondrocytes and the primary ossification center. Scale bar = 200 μm. SO staining of P7 (**e**) and P14 (**f**) hindlimb sections from WT and *Nsd1*^*f/f*^*;Prx1-Cre* mice. Scale bar (top) = 200 μm. Scale bar (bottom) = 500 μm. **g** Whole-mount in situ hybridization (WISH) results for *Col2* in E12.5 embryos (top) and sections of forelimbs (bottom). The dashed purple lines show the digits already present. Scale bar (top) = 500 μm, scale bar (bottom) = 200 μm. **h** SO staining results of E13.5 (top) and E15.5 (bottom) femur sections from WT, *Nsd1*^*f/f*^*;Col2-Cre* mice. The dashed black lines show the borders between hypertrophic chondrocytes and the primary ossification center. Scale bar = 200 μm. SO staining of P7 (**i**) and P14 (**j**) hindlimb sections from WT and *Nsd1*^*f/f*^*;Col2-Cre* mice. Scale bar (top) = 200 μm, Scale bar (bottom) = 500 μm

To explore the functions of NSD1 and histone methylation in chondrogenic differentiation and skeletal growth, we first examined the expression levels of H3K36 methyltransferases in a 3D chondrogenic differentiation system in vitro. Through micromass culture with chondroprogenitor cells,^19^ we collected micromasses at different differentiation times and determined the expression levels of H3K36 methyltransferases. Among the H3K36 methyltransferases, *Nsd1* showed a significantly increased mRNA level (Figs. 1a and S1A), raising the possibility that NSD1 is correlated with chondrogenic differentiation. Then, we detected NSD1 in P7 cartilage and found that NSD1 was widely expressed in articular cartilage and the growth plate (Fig. S1B). Since *Nsd1* germline knockout is embryonic lethal in mice,^13^ we conditionally knocked out *Nsd1* in mesenchymal progenitors or chondrocytes by breeding *Nsd1*^*f/f*^ mice with *Prx1-Cre* or *Col2-Cre* mice, respectively^20,21^ (Fig. S1C, D). The specificity and efficiency of *Nsd1* knockout were verified by qRT-PCR analyses (Fig. S1E, F). In *Nsd1*^*f/f*^*;Prx1-Cre* mice, chondrogenesis lagged behind that in wild-type littermate mice (Fig. 1b), and whole-mount in situ hybridization staining of *Col2* showed delayed autopod formation (Fig. 1c). Safranin O staining results showed retarded formation of both the primary and secondary ossification centers (Fig. 1d–f). However, in *Nsd1*^*f/f*^*;Col2-Cre* mice, chondrogenesis and ossification center formation were normal (Fig. 1g–j). These data indicate that NSD1 deletion in Prx1^+^ mesenchymal progenitors led to embryonic and postnatal limb development defects. However, the above defects were not observed in *Nsd1*^*f/f*^*;Col2-Cre* mice, indicating that NSD1 functions in the very early process of chondrogenesis.

### *Nsd1* deletion in mesenchymal progenitors led to skeletal growth defects in mice

**Fig. 2:**
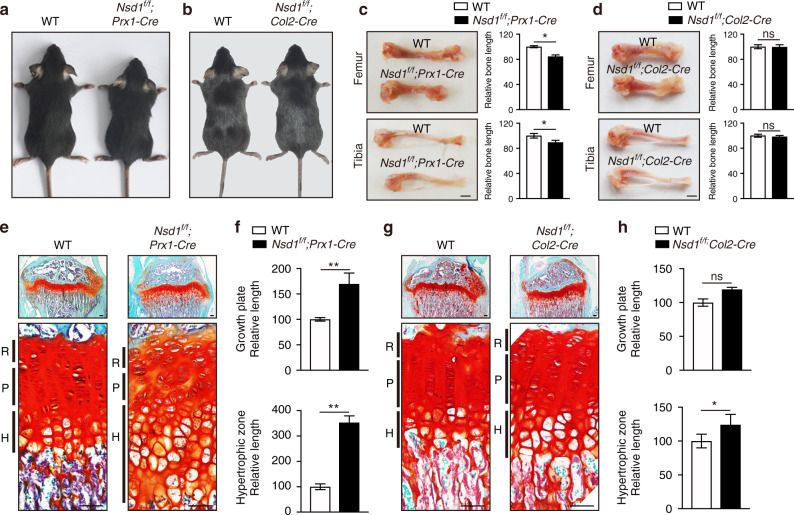
*Nsd1* deficiency in mesenchymal progenitors led to skeletal growth defects in mice. Gross images of 1-month-old WT, *Nsd1*^*f/f*^*;Prx1-Cre* (**a**) and *Nsd1*^*f/f*^*;Col2-Cre* (**b**) mice. Pictures of hindlimbs (left) and quantitative statistics (right) of hindlimb length in 1-month-old WT, *Nsd1*^*f/f*^*;Prx1-Cre* (**c**) and *Nsd1*^*f/f*^*;Col2-Cre* (**d**) mice. Scale bar = 2 mm. The values are presented as the means ± SEMs, *n* = 6. *P < 0.05, ns means not significant. Safranin O (SO) staining results (**e**, **g**) and growth plate quantification data (**f**, **h**) of tibia sections from 1-month-old WT, *Nsd1*^*f/f*^*;Prx1-Cre* (**e**, **f**) and *Nsd1*^*f/f*^*;Col2-Cre* (**g**, **h**) mice. Scale bar (top) = 100 μm. Scale bar (bottom) = 50 μm. The values are presented as the means ± SEMs, *n* = 6. *P < 0.05, **P < 0.01, ns means not significant

Chondrogenic differentiation is a prerequisite for endochondral bone formation, which supports long bone growth. Having seen chondrogenesis and cartilage development defects after NSD1 knockout, we next sought to determine whether these defects impair bone growth. One-month-old *Nsd1*^*f/f*^*;Prx1-Cre* mice showed smaller stature than control mice (Fig. 2a), while *Nsd1*^*f/f*^*;Col2-Cre* mice displayed normal stature (Fig. 2b). *Nsd1*^*f/f*^*;Prx1-Cre* mice displayed shorter hindlimb bone lengths than control mice, whereas the hindlimb bone lengths in *Nsd1*^*f/f*^*;Col2-Cre* mice were comparable to those in control mice (Fig. 2c, d). The growth plate is very important for postnatal bone growth and is the basis of endochondral bone formation. Examination of the growth plate revealed that *Nsd1*^*f/f*^*;Prx1-Cre* mice exhibited thicker growth plates with abnormal cellular morphology in the resting zone, shorter and disorganized columns in the proliferating zone and a strikingly thickened hypertrophic zone (Fig. 2e, f). However, *Nsd1*^*f/f*^*;Col2-Cre* mice only showed slight thickening in the hypertrophic zone of the growth plate (Fig. 2g, h). The bone mass in *Nsd1*^*f/f*^*;Prx1-Cre* mice was lower than that in control mice (Fig. S2A,B), and the bone mass in *Nsd1*^*f/f*^*;Col2-Cre* mice was comparable to that in control mice (Fig. S2C,D). These results show that *Nsd1* knockout in *Prx1*^*+*^ mesenchymal progenitors leads to growth plate malformation and skeletal growth defects in mice, meaning that NSD1 plays an important role during endochondral bone formation.

### *Nsd1* deletion in Prx1^+^ mesenchymal progenitors led to impaired fracture healing in mice

**Fig. 3:**
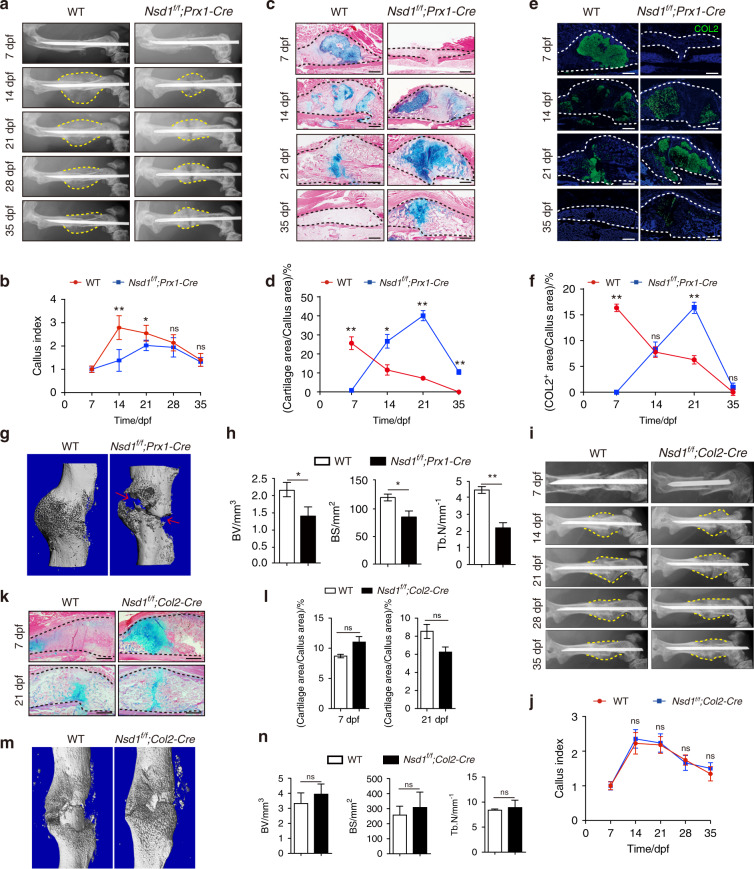
Mice with *Nsd1* knockout in mesenchymal progenitors showed impaired fracture healing. **a** Radiographs of fractured femurs from WT and *Nsd1*^*f/f*^*;Prx1-Cre* mice at different days post fracture (dpf). **b** Quantitative analysis of formed calluses at different days post fracture (dpf). *n* = 5. Alcian blue/eosin staining (**c**) and quantitative results (**d**) of callus sections. The dashed black lines show the location of the callus. Scale bar = 500 μm. *n* = 5. Immunofluorescence staining (**e**) and quantitative results (**f**) of type II collagen in callus sections. The dashed white lines show the location of the callus. Scale bar= 50 µm*. n* = 5. **g** Micro-CT images of calluses in WT and *Nsd1*^*f/f*^*;Prx1-Cre* mice at 18 dpf. **h** Quantitative statistics of micro-CT results of calluses. *n* = 3. **i** Radiographs of fractured femurs in WT and *Nsd1*^*f/f*^*;Col2-Cre* mice at different days post fracture (dpf). **j** Quantitative analysis of formed calluses at different days post fracture (dpf). *n* = 5. Alcian blue/eosin staining (**k**) and quantitative results (**l**) of callus sections. The dashed black lines show the location of the callus. Scale bar = 500 μm. *n* = 5. **m** Micro-CT images of calluses in WT and *Nsd1*^*f/f*^*;Col2-Cre* mice at 21 dpf. **n** Quantitative statistics of micro-CT results of calluses. BV bone volume, BS bone surface, Tb.N trabecular bone number. *n* = 3. The values are presented as the means ± SEMs. *P < 0.05, **P < 0.01, ns means not significant

Bone fracture healing is a regenerative process that recapitulates many skeletal development events, including endochondral and intramembranous ossification.^22^ The chondrogenesis and skeletal growth defects in *Nsd1*^*f/f*^*;Prx1-Cre* mice prompted us to further explore whether the absence of NSD1 affects fracture repair. X-ray scan results showed that *Nsd1*^*f/f*^*;Prx1-Cre* mice had less callus formation than control mice at the same time point (Fig. 3a, b). Histological assessments showed that cartilage formation was delayed in *Nsd1*^*f/f*^*;Prx1-Cre* mice (Fig. 3c, d). Immunofluorescence staining of COL2 also showed delayed cartilage appearance in calluses in *Nsd1*^*f/f*^*;Prx1-Cre* mice during fracture healing (Fig. 3e, f). Micro-CT analysis at 18 days post fracture showed that cracks remained in the calluses only in *Nsd1*^*f/f*^*;Prx1-Cre* mice and not in control mice (Fig. 3g). Quantitative analysis of the micro-CT results showed that the bone volume and trabecular bone number in calluses in *Nsd1*^*f/f*^*;Prx1-Cre* mice were less than those in control mice (Fig. 3h). However, in *Nsd1*^*f/f*^*;Col2-Cre* mice, callus formation was comparable to that in control mice at the same time point (Fig. 3i, j). Alcian blue staining showed normal cartilage formation in *Nsd1*^*f/f*^*;Col2-Cre* mice (Fig. 3k, l). The union of fracture ends was synchronized with that in control mice (Fig. 3m), and the bone formed in the callus showed no difference from that in control mice (Fig. 3n).

These findings suggest that *Nsd1* deletion in Prx1^+^ mesenchymal progenitors leads to impaired fracture healing in mice. Therefore, NSD1 in Prx1^+^ mesenchymal progenitors is indispensable for fracture healing.

### *Nsd1-*deficient chondroprogenitor cells showed decreased chondrogenic differentiation

**Fig. 4:**
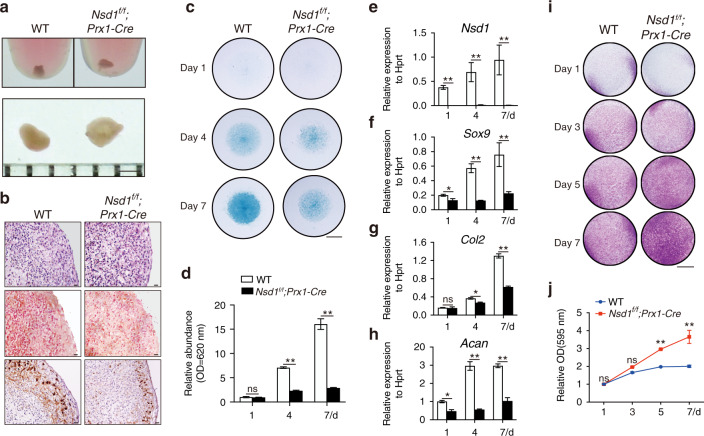
Chondroprogenitor cells with *Nsd1* knockout showed impaired chondrocyte differentiation and increased proliferation. **a** Gross images of pellets formed by chondroprogenitor cells from neonatal mice. Scale bar = 1 mm. **b** HE staining (top), SO staining (middle), and *Col2* in situ hybridization (bottom) results of sections from pellets formed by chondroprogenitor cells. Scale bar = 20 μm. **c** Alcian blue staining results of micromasses cultured for 1, 4, and 7 days with chondroprogenitor cells. Scale bar = 2 mm. **d** Quantitative analysis of Alcian blue staining. The values are presented as the means ± SEMs, *n* = 4. **P < 0.05, ns means not significant. qRT-PCR results for *Nsd1* (**e**) and chondrocyte differentiation marker genes, including *Sox9* (**f**), *Col2* (**g**), and *Acan* (**h**), in micromasses cultured for 1, 4, and 7 days with chondroprogenitor cells. The values are presented as the means ± SEMs, *n* = 4. *P < 0.05, **P < 0.01, ns means not significant. **i** Crystal violet staining results of chondroprogenitor cells cultured for 1, 3, 5, and 7 days. Scale bar = 5 mm. **j** Quantification of crystal violet staining. The values are presented as the means ± SEMs, *n* = 6. **P < 0.01, ns means not significant

To investigate the role of NSD1 in chondrogenic differentiation, we performed 3D pellet culture with chondroprogenitor cells and found that pellets formed by chondroprogenitor cells from *Nsd1*^*f/f*^*;Prx1-Cre* mice were larger and looser (Fig. 4a), with less proteoglycan content and lower *Col2* expression (Fig. 4b) than those formed by chondroprogenitor cells from control mice. Alcian blue staining showed that the proteoglycan content was decreased in micromasses formed by chondroprogenitor cells from *Nsd1*^*f/f*^*;Prx1-Cre* mice (Fig. 4c, d). qRT-PCR analyses confirmed the decreased expression of *Sox9*, *Col2*, and *Acan* in micromasses formed by *Nsd1*-deficient chondroprogenitor cells (Fig. 4e–h). In chondroprogenitor cells, there is a balance among cell differentiation, proliferation, and apoptosis.^17,18^ We next examined the proliferation and apoptosis abilities of NSD1-deficient chondroprogenitor cells. Crystal violet staining, quantification, and the MTT assay revealed increased cell proliferation (Figs. 4i, j and S3A), and the TUNEL assay showed no alterations in the apoptosis (Fig. S3B) of *Nsd1*-deficient chondroprogenitor cells. These data indicate that NSD1 is necessary for chondrogenic differentiation of chondroprogenitor cells.

### *Sox9* was regulated by NSD1 through H3K36 methylation

**Fig. 5:**
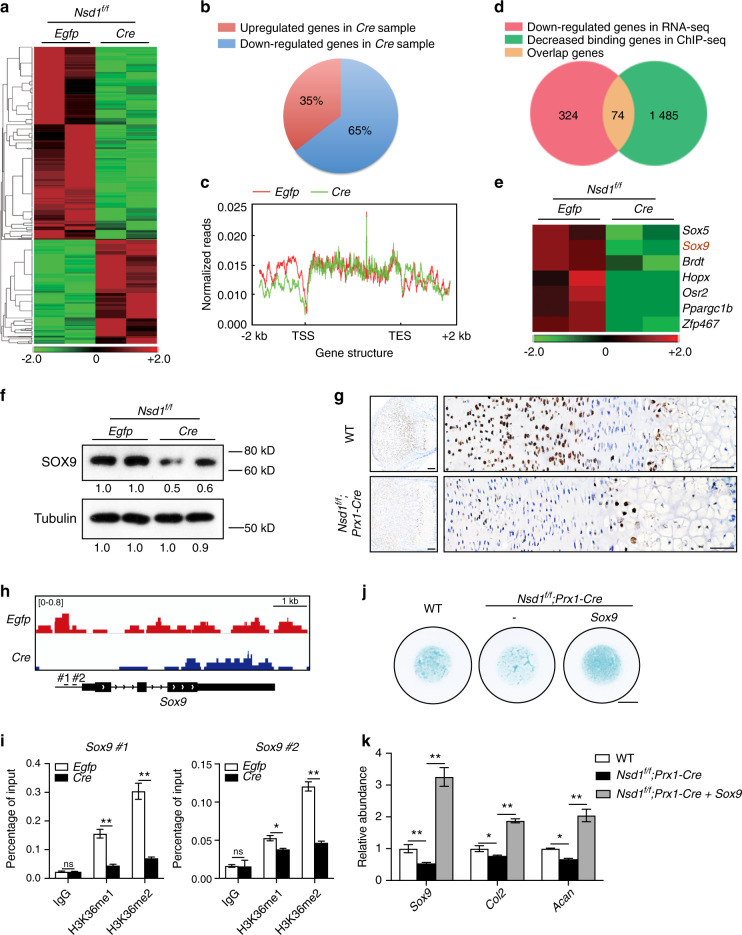
*Sox9* was regulated by NSD1 through H3K36 methylation. **a** Heat map of RNA-seq results for *Egfp*- and *Cre*-expressing immortalized *Nsd1*^*f/f*^ chondroprogenitor cells. **b** Pie chart showing the percentages of differentially expressed genes between *Egfp* and *Cre* samples. **c** Normalized reads of H3K36me2 ChIP-seq analyses in *Egfp*- and *Cre*-expressing immortalized *Nsd1*^*f/f*^ chondroprogenitor cells from 2 kb upstream of the TSS to 2 kb downstream of the TSS in the genome. **d** Venn diagram showing the numbers of genes with decreased expression in RNA-seq data (pink), genes with decreased H3K36me2 occupancy in ChIP-seq data (green), and overlapping genes (yellow). **e** Heat map and annotation of transcription factors from the set of overlapping genes. **f** Western blot analysis of SOX9 in *Egfp*- and *Cre*-expressing immortalized *Nsd1*^*f/f*^ chondroprogenitor cells. **g** Immunohistochemical assay of SOX9 in growth plate sections from P7 mice. Scale bar (left) = 100 μm, scale bar (right) = 5 μm. **h** H3K36me2 binding peaks on *Sox9* in *Egfp*- and *Cre*-expressing immortalized *Nsd1*^*f/f*^ chondroprogenitor cells from the H3K36me2 ChIP-seq assay. **i** ChIP-PCR assay of H3K36me1 (left) and H3K36me2 (right) occupancy of *Sox9*. The values are presented as the means ± SEMs, *n* = 3. *P < 0.05, **P < 0.01, ns means not significant. **j** Alcian blue staining results of micromass culture with chondroprogenitor cells without or with *Sox9* overexpression. Scale bar = 2 mm. **k** qRT-PCR results of *Sox9*, *Col2*, and *Acan* in micromass culture. The values are presented as the means ± SEMs, *n* = 4. *P < 0.05, **P < 0.01

The skeletal growth and fracture healing defects and decreased chondrogenic differentiation in *Nsd1*^*f/f*^*;Prx1-Cre* mice prompted us to examine the underlying mechanisms by which NSD1 regulates chondrogenic differentiation. *Nsd1*^*f/f*^ chondroprogenitor cells were immortalized and infected with lentivirus expressing *Egfp* or *Cre* recombinase. Western blot analysis showed that *Cre* induced depletion of NSD1 and decreased the H3K36me1/2 levels (Fig. S4A). Accordingly, chondrogenic differentiation was impaired in *Cre*-expressing cells (Fig. S4B). RNA sequencing (RNA-seq) data showed that more genes were downregulated (Fig. 5a)—~65% of the differentially expressed genes—than upregulated in *Cre*-expressing cells (Fig. 5b), indicating that H3K36 methylation is mainly linked to the active regulation of transcription.^23,24^ In addition, the H3K36me2 chromatin immunoprecipitation sequencing (ChIP-seq) assay revealed that differential H3K36me2 binding peaks mainly accumulated in promoter regions close to transcription start sites (TSSs) (Fig. 5c). After integration of the H3K36me2 ChIP-seq data with the RNA-seq data, 74 genes showed not only decreased expression levels but also decreased H3K36me2 occupancy in *Cre*-expressing cells (Fig. 5d). Gene Ontology (GO) analysis revealed that these genes were mainly involved in cell differentiation (Fig. S5). Among these genes were seven transcription factors, including *Sox9*, the key transcription factor for chondrogenic differentiation (Fig. 5e). SOX9 expression was decreased in both *Cre*-expressing cells and the growth plate of *Nsd1*^*f/f*^*;Prx1-Cre* mice (Fig. 5f, g). Mice with *Nsd1* knockout in chondrocytes did not show a change in the SOX9 protein level (Fig. S6A). H3K36me2 ChIP-seq data showed decreased H3K36me2 levels on the promoter of *Sox9* in *Cre*-expressing cells (Fig. 5h). ChIP-PCR assays confirmed the decreased occupancy of H3K36me1 and H3K36me2 in the promoter region of *Sox9* in NSD1 knockout cells (Fig. 5i). Moreover, overexpression of *Sox9* in chondroprogenitor cells rescued the chondrogenic differentiation defects of *Nsd1-*deficient cells, as demonstrated by Alcian blue staining and qRT-PCR analysis of chondrogenic differentiation marker genes (Fig. 5j, k). Collectively, the above data indicate that the regulation of gene expression by NSD1 occurs mainly through H3K36 methylation in the TSS region and that *Sox9* is directly regulated by NSD1 through H3K36me1/2 occupancy in the *Sox9* promoter region.

### NSD1 showed direct regulation on *Hif1α*

**Fig. 6:**
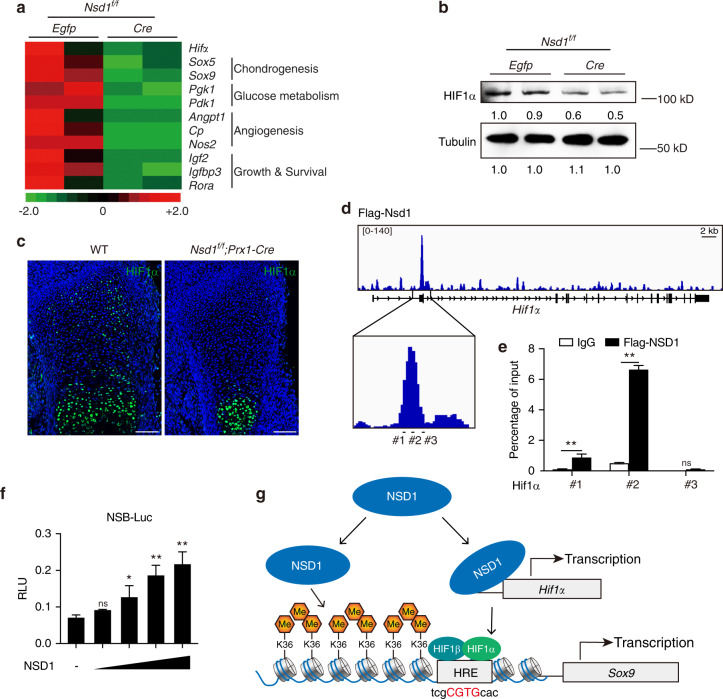
NSD1 directly regulated *Hif1α*. **a** Heat map of *Hif1α* and its target genes from the RNA-seq results of *Egfp*- and *Cre*-expressing immortalized *Nsd1*^*f/f*^ chondroprogenitor cells. **b** Western blot analysis of the HIF1α level in *Egfp*- and *Cre*-expressing immortalized *Nsd1*^*f/f*^ chondroprogenitor cells. **c** Immunofluorescence analysis of HIF1α in limb buds of E15.5 mice. Scale bar = 100 μm. **d** NSD1 binding peaks on *Hif1α* in ATDC5 cells from the Flag-NSD1 ChIP-seq assay. **e** ChIP-PCR assay of NSD1 binding on *Hif1α*. The values are presented as the means ± SEMs, *n* = 3. **P < 0.01, ns means not significant. **f** Luciferase assay of the NSD1-specific binding (NSB) region in the *Hif1α* promoter in C3H10 cells treated with NSD1. The values are presented as the means ± SEMs, *n* = 3. *P < 0.05, **P < 0.01, ns means not significant. **g** Model that summarizes our findings on the role of NSD1 in regulating *Sox9* directly and indirectly. On the one hand, NSD1 can directly promote *Sox9* expression by regulating the levels of H3K36me1/2 in the *Sox9* promoter region. On the other hand, NSD1 directly binds to the promoter region of *Hif1α*, activating *Hif1α* transcription and ultimately promoting *Sox9* expression

As the key regulator of chondrogenic differentiation, *Sox9* is regulated by a number of factors, including HIF1α.^4^ When RNA-seq data were analyzed separately, we found that the levels of *Hif1α* and its target genes were decreased after *Nsd1* deletion (Figs. 6a and S7A). Western blot analysis and immunofluorescence staining showed decreased protein levels of HIF1α after *Nsd1* knockout in mesenchymal progenitors (Fig. 6b, c), and no change in the HIF1α protein level occurred after *Nsd1* knockout in chondrocytes (Fig. S8A). The H3K36me2 ChIP-seq results showed no obvious binding peak differences in *Hif1α* (Fig. S9A); thus, we performed NSD1 ChIP-seq with an anti-Flag antibody after transfecting the Flag-NSD1 plasmid into ATDC5 cells, a chondrogenic cell line.^25^ From the Flag-NSD1 ChIP-seq results, we observed a specific NSD1 binding peak in the *Hif1α* promoter region (Fig. 6d). The ChIP-PCR assay results verified this binding (Fig. 6e). Next, we cloned the genomic sequence of the NSD1-specific-binding (NSB) peak into the pGL3 luciferase reporter (NSB-Luc) plasmid and assessed the effects of NSD1 on this reporter. The luciferase reporter assay showed that NSD1 can activate NSB-Luc (Fig. 6f), indicating positive regulation of *Hif1α*. Since *Sox9* is a well-known target gene regulated by HIF1α, these data indicate that NSD1 directly regulates *Hif1α* and that the regulation of *Sox9* by NSD1 can also be achieved indirectly through *Hif1α* (Fig. 6g).

## Discussion

In this study, we found that the histone methyltransferase NSD1 plays a key role in chondrogenic differentiation. We observed increased *Nsd1* mRNA levels during chondrogenic differentiation. *Nsd1*^*f/f*^*;Prx1-Cre* mice showed delayed chondrogenesis, delayed primary and secondary ossification center formation, shorter stature, and malformation of the growth plate, but these phenotypes were not seen in *Nsd1*^*f/f*^*;Col2-Cre* mice, meaning that NSD1 mainly functions in the stage before Col2^+^ chondrocyte formation. From single-cell RNA-seq data of E11.5 limb buds, we found that the distribution of *Nsd1* was more overlapped with that of *Prrx1* and broader than that of *Col2a1* (Fig. S10A).^26^ The skeletal growth defects in *Nsd1*^*f/f*^*;Prx1-Cre* mice were due to aberrant growth plate formation, especially the abnormal resting zone and disorganized proliferating zone, consistent with a previous finding that chondrocyte progenitors in the resting zone can supply cells for longitudinal bone growth in postnatal mice.^27^ In addition to participating in bone formation and elongation under physiological conditions, chondrogenic differentiation also participates in fracture healing under pathological conditions.^28^ In the fracture model, mice with NSD1 deletion showed impaired fracture healing, delayed appearance of cartilage, and decreased endochondral bone formation. Further study showed that NSD1 deletion disrupted the balance between the proliferation and differentiation of chondroprogenitor cells.

In *Nsd1*^*f/f*^*;Prx1-Cre* mice, we observed shorter stature and decreased bone length, inconsistent with the pre- and postnatal overgrowth in Sotos syndrome patients.^16^ In NSD1 heterozygous mice, the growth rate was normal, and the Sotos phenotype was only observed with careful analysis of the growth pattern, which was more subtle than that in humans.^13^ This inconsistency is also observed in Df(13)Ms2Dja (+/−) mice, a chromosome-engineered mouse model of Sotos syndrome; most of the Sotos phenotypes, except for overgrowth, are replicated in these mice, and Df(13)Ms2Dja (+/−) mice show reduced gestational and postnatal growth.^29^ In this study, the inconsistency in bone growth between mice and humans may be attributed to the deletion of NSD1 within a specific cell population at a particular stage of development in our mouse model and the observation that NSD1 may play divergent roles in regulating bone growth in mice and humans. Overgrowth-related genes identified in patients do not always cause overgrowth in mice. For example, mice carrying DNMT3A mutations show postnatal growth retardation, which is different from the phenotype of *DNMT3A* overgrowth syndrome patients.^30,31^ In addition, deletion of EZH1 and EZH2 in chondrocytes causes severe skeletal growth impairment in mice, which is due to reduced growth plate chondrogenesis rather than longitudinal bone overgrowth.^12,32^

Over the past decades, the study of NSD1 has mainly focused on its function in tumorigenesis, including in head and neck squamous cell carcinomas,^33^ laryngeal tumors,^34^ myelodysplastic syndromes,^35^ and so on. H3K36 methylation is also related to tumor formation, and H3K36M mutation impairs the differentiation potential of mesenchymal progenitors and leads to undifferentiated sarcoma generation.^18^ H3K36M leads to decreased H3K36 di- and trimethylation, activating cancer pathways and resulting in chondroblastoma.^17^ A recent study found that NSD1-mediated H3K36me2 is required for the maintenance of DNA methylation at intergenic regions, which is crucial for the regulation of downstream gene expression.^36^ Collectively, these findings indicate that NSD1-mediated histone modification plays important roles in various pathophysiological processes. In our study, *Nsd1* knockout led to a decrease in H3K36me1 and H3K36me2, leading to defects in chondrogenesis and growth plate formation. However, our previous study demonstrated that there was no cartilage phenotype in *Setd2*^*f/f*^*;Prx1-Cre* mice.^37^ As SET domain-containing protein 2 (SETD2) is the only methyltransferase for H3K36me3, our current study suggested the different functions of different forms of H3K36 methylation. We performed RNA-seq and ChIP-seq analysis and found that NSD1 and H3K36 methylation regulate the transcription of different sets of genes. Among these genes, SOX9 can promote chondrogenic differentiation. It has been proven that SOX9 is indispensable for skeletogenesis, especially for growth plate formation.^2,3,38,39^ The expression of *Sox9* was regulated by NSD1 through H3K36me1 and H3K36me2 occupancy of the promoter (Fig. 5), and overexpression of *Sox9* rescued the chondrogenic differentiation impairment (Fig. 5), suggesting that NSD1 is a key epigenetic regulator of chondrogenesis, at least partially through the regulation of *Sox9* expression. NSD1 deficiency in *Prx1*-positive MSCs affected limb formation, with abnormal *Col2*-positive chondrocyte formation and abnormal *Sox9* expression (Figs. 1c and 5g). However, *Nsd1*^*f/f*^;*Col2-Cre* mice had normal limb formation (Fig. 1g) with normal *Sox9* expression (Fig. S6A), suggesting that NSD1 functions before the activation of *Col2-cre* or the expression of collagen II. In summary, we believe that NSD1 functions as an epigenetic regulator of *Sox9* expression mainly in *Col2-cre*-negative chondroprogenitor cells but not in *Col2-cre*-positive chondrocytes.

Moreover, we found from the transcriptome analysis that the expression of *Hif1α* and its target genes was reduced when *Nsd1* was depleted. HIF1α plays a crucial role during chondrogenic differentiation and limb development. Mice with limb bud mesenchyme-specific *Hif1a* knockout show significantly shorter hindlimbs with abnormal cartilage formation and decreased differentiation of prechondrogenic cells through direct regulation of *Sox9.*^4^ It has been known for years that histone lysine methyltransferases (KMTs) can promote or inhibit gene expression by targeting the enhancer or promoter regions of different genes.^40^ In addition, some KMTs can regulate target gene expression independent of HMT activity. EZH2 can promote cyclin D1 expression directly in natural killer cells independent of its enzymatic activity.^41^ G9a, another histone KMT, inhibits adipogenesis by repressing *Pparγ* expression in a manner dependent on its HMT activity and promoting *Wnt10a* expression in an enzymatic activity-independent manner.^42^ Here, H3K36me2 occupancy on *Hif1α* showed no difference after NSD1 knockout, and among the seven transcription factors found by combined analysis of the RNA-seq and H3K36me2 ChIP-seq data, only *Sox9*, *Hopx*, *Osr2*, and *Zfp467* showed obvious NSD1 binding peaks (Fig. S9B–H), revealing that NSD1 binding and H3K36me2 occupancy on target genes are not entirely synchronous. In this study, NSD1 bound to the *Hif1α* promoter directly and activated *Hif1α* transcription, raising the possibility that NSD1 may also function independent of HMT activity.

Collectively, we identified NSD1 as a novel regulator of chondroprogenitor cell fate and suggested that epigenetic regulation of SOX9 by NSD1 is an important process for chondrogenesis. These findings suggest that modulation of NSD1 and H3K36 methylation would have therapeutic potential for skeletal growth defects and fracture healing disorders resulting from chondrogenic differentiation impairment. Revealing the function of NSD1 in chondrogenic differentiation and bone growth is helpful to understand the overgrowth of Sotos syndrome patients with *NSD1* mutations and to expand our understanding of the function of epigenetic regulation in chondrogenesis and skeletal biology.

## Materials and methods

### Ethics statement

All animal experiments were conducted in accordance with a protocol approved by the Animal Care and Use Committee of Shanghai Institute of Biochemistry and Cell Biology, Chinese Academy of Sciences (approval number: SIBCB-NAF-14-001-S350-019). Animals were bred and maintained under specific pathogen-free conditions in the institutional animal facility of the Shanghai Institute of Biochemistry and Cell Biology, Chinese Academy of Sciences.

### Mice

*Nsd1*^*f/f*^ mice were purchased from the Jackson Laboratory. The *Prx1-Cre* mouse strain was a gift from Andrew McMahon. The *Col2-Cre* mice were kindly provided by Dr. Xiao Yang. All mice analyzed were maintained on the C57BL/6 background.

### Assessment of *Nsd1* knockout efficiency

The *Nsd1* gene knockout efficiency assay was performed in three tissues: cartilage, bone, and liver. Cartilage was taken from the tibial plateau, and bone was obtained by cutting out the ends of the tibial growth plate and flushing out the bone marrow. Liver tissue was used as the negative control.

### Mouse femoral fracture

The fracture model was established as described previously with 6-week-old mice.^43^ Weekly radiographs were performed on mice with fractures to measure the repair process with a Faxitron Model MX-20 instrument (Faxitron, America). The callus index was defined as the maximum diameter of the callus divided by the diameter of the bone.

### X-ray analysis

Prior to X-ray analysis, mice were anesthetized with 2% chloral hydrate solution (10 μL·g^−1^ body weight) injected intraperitoneally. Fractures were confirmed and monitored weekly under anesthesia using a Faxitron MX-20 Cabinet X-ray System (Faxitron X-ray Corp.).

### Micro-CT analysis

For micro-CT analysis, soft tissue was removed from fractured femurs from age- and sex-matched mice, and the femurs were fixed with 70% ethanol. Fractured femurs from *Nsd1*^*f/f*^*;Prx1-Cre* mice and 1-month-old *Nsd1*^*f/f*^*;Col2-Cre* mice were scanned with a Scanco Micro CT80 instrument (SCANCO Medical, Switzerland) at a resolution of 10 μm. Fractured femurs from *Nsd1*^*f/f*^*;Col2-Cre* mice and 1-month-old *Nsd1*^*f/f*^*;Prx1-Cre* mice were scanned with a Skyscan 1176 scanner (Bruker, Kartuizersweg, Belgium) at a spatial resolution of 9 μm. For statistical analysis of trabecular bone in the callus, the whole region of the callus with a threshold of 85–255 was used. A Gaussian noise filter optimized for murine bones was applied to reduce the noise in the thresholded 2D image, and 3D images were reconstructed.^44^ Indices of trabecular and cortical bone are shown according to the guidelines.^45^

### Cell culture

Chondroprogenitor cells were obtained from the femoral condyles and tibial plateau of newborn mice. The cartilage was digested with 1 mg·mL^−1^ collagenase II (Sigma, C6885) for 2 h at 37 °C, and the digests were discarded. The remaining tissue was digested with half the concentration of collagenase II overnight at 37 °C, and the digests were filtered through a 70 μm cell strainer (Falcon, 352350) the next day. Cells were plated in α-MEM (Corning, 10-022-CVR) supplemented with 10% fetal bovine serum (FBS) and 1% penicillin/streptomycin. ATDC5 cells were cultured in DMEM:F12 (1:1) supplemented with 5% FBS and 1% penicillin/streptomycin. C3H10 cells were cultured in α-MEM (low glucose) supplemented with 10% FBS and 1% penicillin/streptomycin.

### Micromass culture

Micromass culture was performed when chondroprogenitor cells were 80%–90% confluent. Chondroprogenitor cells were digested, resuspended at 1 × 10^7^ cells per cell, and plated in a 12.5 μL droplet of cell suspension in the center of a 12-well-plate; the plate was placed at 37 °C for 2 h, and chondrogenic differentiation medium, which contained DMEM (Corning, 10-013-CVR), 10 ng·mL^−1^ TGFβ3 (Peprotech, 100-36E), 100 nmol·L^−1^ dexamethasone (Sigma, D1756), 50 μg·mL^−1^ L-ascorbic acid 2-phosphate (Sigma, A8960), 1 mmol·L^−1^ sodium pyruvate (Sigma, 25-000-CIR), 40 μg·mL^−1^ proline (Sigma, P5607), and 1% ITS (Cyagen, ITSS-10201-10), was then gently added. At different time points, micromasses were acidified with 0.1 N HCl and were then stained with 1% Alcian blue (Sigma, A5268). Quantification of Alcian blue staining was performed by measuring the absorbance at 620 nm after dissolving the stained micromass with 6 M guanidine hydrochloride solution.

### Pellet culture

Pellet culture was performed when chondroprogenitor cells were 80%–90% confluent. Chondroprogenitor cells were digested and resuspended at 1 × 10^7^ cells per mL, 12.5 μL of cell suspension was added to 500 μL of chondrogenic differentiation medium in a 15 mL tube, the tube was centrifuged at 400 × *g* for 4 min to pellet the cells in the bottom of tube, the tube was allowed to stand, and cells were cultured at 37 °C. The culture medium was replaced with fresh medium every 3 days in the first week and weekly thereafter.

### Immortalization of chondroprogenitor cells

Chondroprogenitor cells were infected with pLenti-CMV-SV40 lentivirus expressing simian virus 40 (SV40) T antigen to achieve immortalization.

### Lentiviruses and infection

Lentiviral vectors expressing *Egfp* and *Cre* were constructed by inserting the genes’ CDSs into the pLenti vector. Virus packaging was conducted according to the VSVG-delta 8.9 system. Mouse chondroprogenitor cells were cultured for 2 days, infected with lentivirus for 24 h, and treated with puromycin for 48 h.

### Histology and immunohistochemistry

Hindlimbs and fractured femurs from mice were fixed with 4% paraformaldehyde for 48 h at 4 °C, decalcified in 10% EDTA, and embedded in paraffin. Each sample was sectioned sagittally at a thickness of 8 μm for staining. HE staining and Safranin O staining were performed. Immunohistochemical staining was conducted using a standard protocol. The in situ hybridization probe for *Col2* was a gift from the Laurie H. Glimcher Laboratory.

### Immunofluorescence

Sections were blocked in PBS with 10% horse serum for 1 h and were then stained overnight with a specific antibody at 4 °C. Secondary antibodies were used according to the species of the primary antibody. DAPI (Sigma, D8417) was used for counterstaining. Slides were mounted with anti-fluorescence quenching mounting medium (Dako, S3023), and images were acquired with an Olympus BX51 microscope.

### Antibodies

Antibodies specific for the following molecules were used: NSD1 (Bioss, bs-8170R), COL2 (Abcam, ab34712), H3K36me1 (Abcam, ab9048), H3K36me2 (Abcam, ab9049), H3K36me3 (Abcam, ab9050), SOX9 (Millipore, AB5535), HIF1α (WB: Novus, NB100-134; IF: Bioss, bs-0737R), and Flag (Sigma, F1804).

### Western blot analysis

Cells were harvested and lysed with EBC buffer (1% NP-40, 10% glycerol, 135 nmol·L^−1^ NaCl, 20 mmol·L^−1^ Tris (pH 8.0)) containing a protease inhibitor (MCE, HY-K0010). Then, lysates were separated through SDS-PAGE and transferred to a PVDF membrane (Bio-Rad, 1620177). After incubation with specific antibodies, we used an enhanced chemiluminescence kit (Millipore, P90720) to detect protein signals. Quantitative data were analyzed by ImageJ software (Bethesda, MD, USA).

### RNA-seq and data processing

*Egfp-* and *Cre*-expressing immortalized *Nsd1*^*f/f*^ chondroprogenitor cells were collected, and total RNA was extracted with TRIzol Reagent (Sigma, T9424). High-throughput sequencing was performed by the Computational Biology Omics Core, CAS-MPG Partner Institute for Computer Biology (PICB), Shanghai Institutes for Biological Sciences, Chinese Academy of Sciences. Raw reads were mapped to the mm10 genome using the TopHat program. We assigned each gene an expression value in fragments per kilobase per million using Cufflinks software. Then, Cuffdiff software was used to identify differentially expressed genes between *Egfp-* and *Cre*-expressing samples. Differentially expressed gene heat maps were clustered by k-means clustering using the Euclidean distance as the distance and visualized using Heml software. GO analysis was carried out with the DAVID Functional Annotation Bioinformatics Microarray Analysis tool.

### Real-time PCR analysis

Total RNA was isolated from different tissues and cells with TRIzol Reagent (Sigma, T9424) and reverse-transcribed with a PrimeScript RT Reagent Kit (Takara, RR037A). Real-time reverse transcription-PCR was performed in a Bio-Rad CFX Connect Real-Time System. The primer sets used were *Nsd1*: sense AAACTCGGAGGGTGCT, anti-sense CCTGAGGCGTTTCTTCT; *Nsd2*: sense TGCCAAAAAGGAGTACGTGTG, anti-sense CTTCGGGAAAGTCCAAGGCAG; *Nsd3*: sense TCCACTGGTGTTAAGTTCCAGG, anti-sense GGCACCTCTTGTGTTAATTTTGG; *Setd2*: sense AAATCAGGTACTGGGGCTACA, anti-sense GGCCCATTTCATTAGATCAGGGA; *Ash1l*: sense CCTCGGTGGACTAAAGTGGTG, anti-sense CGCTGGCTCAGAACTATTTGA; *Smyd2*: sense AAGGATTGTCAAAATGTGGACGG, anti-sense ATGGAGGAGCATTCCAGCTTG; *Col2*: sense CGGTCCTACGGTGTCAGG, anti-sense GCAGAGGACATTCCCAGTGT; *Sox9*: sense TTCCTCCTCCCGGCATGAGTG, anti-sense CAACTTTGCCAGCTTGCACG; *Acan*: sense AATCCCCAAATCCCTCATAC, anti-sense CTTAGTCCACCCCTCCTCAC; *Hif1α*: sense AGATCTCGGCGAAGCAAAGAGT, anti-sense CGGCATCCAGAAGTTTTCTCACAC; *Sox5*: sense CCCGTGATCCAGAGCACTTAC, anti-sense CCGCAATGTGGTTTTCGCT; *Pgk1*: sense ATGTCGCTTTCCAACAAGCTG, anti-sense GCTCCATTGTCCAAGCAGAAT; *Pdk1*: sense GGACTTCGGGTCAGTGAATGC, anti-sense TCCTGAGAAGATTGTCGGGGA; *Angpt1*: sense CACATAGGGTGCAGCAACCA, anti-sense CGTCGTGTTCTGGAAGAATGA; *Cp*: sense CTTAGCCTTGGCAAGAGATAAGC, anti-sense GGCCTAAAAACCCTAGCCAGG; *Nos2*: sense GTTCTCAGCCCAACAATACAAGA, anti-sense GTGGACGGGTCGATGTCAC; *Igf2*: sense GTGCTGCATCGCTGCTTAC, anti-sense ACGTCCCTCTCGGACTTGG; *Igfbp3*: sense CCAGGAAACATCAGTGAGTCC, anti-sense GGATGGAACTTGGAATCGGTCA; *Rora*: sense GTGGAGACAAATCGTCAGGAAT, anti-sense TGGTCCGATCAATCAAACAGTTC; and *Hprt*: sense GTTAAGCAGTACAGCCCCAAA, anti-sense AGGGCATATCCAACAACAAACTT.

### ChIP-seq and ChIP-PCR

Cells were fixed with 1% formaldehyde for 10 min, and the crosslinking reaction was terminated with glycine for 5 min (final concentration = 0.125 mol·L^−1^). After two washes with precooled PBS (containing a protease inhibitor), the cells were removed by scraping and resuspended in SDS lysis buffer (50 mmol·L^−1^ Tris-HCl (pH 7.5), 10 mmol·L^−1^ EDTA, 1% SDS, and protease inhibitor) and sonicated. Cells were centrifuged to obtain cell extracts, which were then added to precleaning protein G agarose and rotated for 1 h at 4 °C. Extracts were centrifuged, and supernatants were harvested into new tubes. ChIP assays were performed using H3K36me1/2 or Flag antibodies. Normal IgG was used as negative control. ChIP-PCR was used to amplify various genomic regions of the target gene, and the primers used were *Sox9* #1: sense GACTCCAGGCGCAGAAGCCC, anti-sense CCGGGACTTCGCTGGCGTTT; *Sox9* #2: sense CACATCGGTTCACACGGAGA, anti-sense GTGGGGTGAGGGGACTTGGA. *Hif1α* #1: sense CTCGGCTTTTCCCTCCCC, anti-sense AGTCCTCGCGTCCCCTCA; *Hif1α* #2: sense GGGCAGTGTCTAGCCAGGC, anti-sense AAGTCCAGAGGCGGGGTG; and *Hif1α* #3: sense CGGTCCACGTCGCCATC, anti-sense CGGGAGCTAGAGGCGTAC. For the ChIP-PCR assay of micromasses harvested at different time points, the collected micromasses were cut into very small pieces after fixation and termination. Sonication was carried out with a QSONICA Q800R with a 30% sonicator amplitude, a schedule of 10 s on and 10 s off, and a total sonication (“on”) time of 30 min. Subsequent steps were consistent with those used for the ChIP-PCR assay of cells.

### ChIP-seq data processing

High-throughput sequencing was performed by the Computational Biology Omics Core, CAS-MPG PICB, Shanghai Institutes for Biological Sciences, Chinese Academy of Sciences. The SOAP alignment tool was used to align the ChIP-seq reads to the mouse genome build mm10. Reads with fewer than two mismatches that uniquely mapped to the genome were used in subsequent analyses. We calculated the distance from the peak centers to the annotated TSSs and then defined the nearest genes as peak-related genes.

### Transient transfections and reporter gene assays

For transient transfections, C3H10 cells were seeded overnight in a 12-well plate at a concentration of 5 × 10^4^ cells per well. Cells were then transfected with the *Hif1α*-Luc or HRE-Luc reporter plasmid and various combinations of NSD1 and HIF1A expression constructs as indicated. Forty-eight hours after transfection, luciferase assays were performed using the Dual-Luciferase Reporter Assay System (Promega). The *Hif1α*-Luc plasmid was constructed by inserting KpnI/XhoI-digested PCR products, which were amplified using the forward primer 5′-GGggtaccGGGCAGTGTCTAGCCAGGC-3′ and reverse primer 5′-CCGctcgagAAGTCCAGAGGCGGGGTG-3′, into the KpnI/XhoI-digested pGL3-Basic luciferase reporter plasmid.

### Statistical analysis

Quantitative data are presented as the mean ± SEM values as indicated. The statistical significance of differences between WT and CKO mice was evaluated with GraphPad using unpaired two-tailed Student’s *t* tests, and one-way ANOVA was used to detect the effects of *Sox9* treatment. *P* < 0.05 was considered statistically significant. The number of samples shown in each figure legend is the number of biological replicates. Three technical replicates were used for each experiment.

### MTT assay

The MTT cell viability assay was conducted following the instructions provided in the MTT Cell Proliferation Assay Kit (Sangon Biotech, E606334).

### TUNEL assay

The TUNEL apoptosis assay was conducted on paraffin sections following the instructions provided in the DeadEnd™ Fluorometric TUNEL System (Promega, G3250).

## Supplementary information

Supplementary Figures

Supplementary Information
