# Correction: H3K36 methyltransferase NSD1 regulates chondrocyte differentiation for skeletal development and fracture repair

**DOI:** 10.1038/s41413-021-00160-2

**Published:** 2021-07-26

**Authors:** Rui Shao, Zhong Zhang, Zhan Xu, Huiling Ouyang, Lijun Wang, Hongwei Ouyang, Matthew Greenblatt, Xi Chen, Weiguo Zou

**Affiliations:** 1 Shanghai Institute of Microsurgery on Extremities, and Department of Orthopedic Surgery, Shanghai Jiao Tong University Affiliated Sixth People’s Hospital, Shanghai, China; 2 State Key Laboratory of Cell Biology, Shanghai Institute of Biochemistry and Cell Biology, CAS Center for Excellence in Molecular Cell Sciences, Chinese Academy of Sciences, University of Chinese Academy of Sciences, Shanghai, China; 3 Dr. Li Dak Sum & Yip Yio Chin Center for Stem Cell and Regenerative Medicine, Zhejiang University-University of Edinburgh Institute, Zhejiang University, Hangzhou, Zhejiang China; 4 China Orthopedic Regenerative Medicine Group, Hangzhou, Zhejiang China; 5 Department of Pathology and Laboratory Medicine, Weill Cornell Medicine, New York, NY USA; 6 Department of Molecular and Cellular Biology, Lester and Sue Smith Breast Center, Dan L. Duncan Cancer Center, Baylor College of Medicine, Houston, TX USA

**Keywords:** Bone, Pathogenesis

Correction to: *Bone Research* 10.1038/s41413-021-00148-y, published online 7 June 2021

During a reread of our article published in Bone Research^1^, we regrettably found that the title of Fig. 3k was labeled incorrectly during the production of the paper. It should be *Nsd1*^*f/f*^*;Col2-Cre*, consistent with the figure legends. We sincerely apologize for this mistake.
