# Supplementary material for: H3K36 methyltransferase NSD1 regulates chondrocyte differentiation for skeletal development and fracture repair
Source: Bone Res. 2021 Jun 7;9:30. doi: 10.1038/s41413-021-00148-y (PMC8185073; doi:10.1038/s41413-021-00148-y)
Supplement: Supplementary file 1 — Supplementary Figures [file 41413_2021_148_MOESM1_ESM.docx]

**Supplementary Figures**

**Supplemental figure 1. Generation of *Nsd1* conditional knockout mice.**

**(A)** Absolute expression levels of *Nsd1*, *Nsd2* and *Nsd3* during micromass culture. The values are presented as the means ± SEMs, n=6. *P<0.05, **P<0.01, ns means not significant.

**(B)** Immunohistochemical assay of NSD1 in cartilage sections from p7 mice. Scale bar (left) = 200 µm.

**(C-D)** Strategy for *Nsd1*-specific deletion in Prx1-expressing **(C)** and Col2 expressing **(D)** cells. The structure of the *Nsd1* allele and the LoxP site are illustrated. Homologous recombination causes deletion of exon 6 (E6).

**(E-F)** qRT-PCR results of *Nsd1* in different tissues. The values are presented as the means ± SEMs, n=6. *P<0.05, **P<0.01, ns means not significant.

**Supplemental figure 2. Mice with Nsd1 knockout in chondroprogenitor cells showed decreased bone mass.**

**(A)** Coronal section images of 3D (top)and 2D (bottom) micro-CT scans of femurs isolated from 4-week-old WT and *Nsd1^f/f^;Prx1-Cre* mice. The red asterisk indicates the primary spongiosa, and the green asterisk indicates the secondary spongiosa.

**(B)** Quantitative analysis of micro-CT data for femurs isolated from 4-week-old WT and *Nsd1^f/f^;Prx1-Cre* mice. Bone volume fraction (BV/TV), bone surface density (BS/TV), trabecular number (Tb.N), trabecular thickness (Tb.Th) and trabecular spacing (Tb.Sp). The values are presented as the means ± SEMs, n=6. *P<0.05, **P<0.01, ns means not significant.

**(C-D)** Coronal section images **(C)** and quantitative analysis **(D)** of micro-CT data for femurs isolated from 5-week-old WT and *Nsd1f/f;Col2-Cre* mice. The values are presented as the means ± SEMs, n=6. ns means not significant.

**Supplemental figure 3. Nsd1 knockout in chondroprogenitor cells did not affect apoptosis in cartilage.**

**(A)** MTT assay of primary chondroprogenitor cells from WT and *Nsd1^f/f^;Prx1-Cre* mice. The values are presented as the means ± SEMs, n=6. ** P<0.01.

**(B)** TUNEL assay results showing TUNEL signals (green) in E15.5 limb buds. Scale bar =100 μm.

**Supplemental figure 4. Properties of Egfp- and Cre-expressing immortalized *Nsd1^f/f^* chondroprogenitor cells.**

**(A)** Western blot analysis of NSD1 and H3K36me1/2 levels in Egfp- and Cre-expressing immortalized *Nsd1^f/f^* chondroprogenitor cells.

**(B)** Alcian blue staining results of micromass culture with Egfp- and Cre-expressing immortalized *Nsd1^f/f^* chondroprogenitor cells. Scale bar = 2 mm.

**Supplemental figure 5. Gene ontology (GO) analysis of genes with decreased expression identified by RNA-seq.**

**(A-C)** GO-BP (biological pathway) **(A)**, GO-MF (molecular function) **(B)** and KEGG pathway **(C)** analysis results of the genes with downregulated expression identified by RNA-seq.

**Supplemental figure 6. Nsd1 knockout in Col2-positive chondrocytes did not affect the level of SOX9.**

**(A)** Immunohistochemical analysis of SOX9 in limbs from WT and *Nsd1f/f;Col2-Cre* mice. Scale bar =250 μm.

**Supplemental figure 7. Deletion of Nsd1 in mesenchymal progenitors led to decreased expression of *Hif1α* and conventional hypoxia-responsive genes.**

**(A)** The mRNA levels of *Hif1α* and its target genes were determined by qRT-PCR in Egfp- and Cre-expressing immortalized chondroprogenitor cells. The values are presented as the means ± SEMs, n=6. * P<0.05, **P<0.01.

**Supplemental figure 8. Nsd1 knockout in Col2-positive chondrocytes did not affect the**

**level of HIF1α.**

**(A)** Immunofluorescence analysis of HIF1α in limb buds from E15.5 mice. Scale bar = 100 μm.

**Supplemental figure 9. H3K36me2/NSD1 binding peaks on transcription factors downregulated in Nsd1-deficient cells.**

**(A)** H3K36me2 binding peaks on Hif1α in Egfp- and Cre-expressing immortalized chondroprogenitor cells from the H3K36me2 ChIP-seq assay.

**(B-H)** NSD1 binding peaks on *Sox5* **(B)**, *Sox9* **(C)**, *Brdt* **(D)**, *Hopx* **(E)**, *Osr2* **(F)**, *Ppargc1b* **(G)** and *Zfp467* **(H)** in ATDC5 cells from the Flag-NSD1 ChIP-seq assay.

**Supplemental figure 10. Distribution of *Prrx1*, *Col2a1* and *Nsd1* expression in the sc-RNA-seq data from E11.5 limb buds.**

**(A)** Distribution of *Prrx1*, *Col2a1* and *Nsd1* expression in the single-cell RNA-seq data from E11.5 limb buds.
