# Supplementary figures and images for: H3K36 methyltransferase NSD1 regulates chondrocyte differentiation for skeletal development and fracture repair
Source: Bone Res. 2021 Jun 7;9:30. doi: 10.1038/s41413-021-00148-y (PMC8185073; doi:10.1038/s41413-021-00148-y)

**Fig. S1**

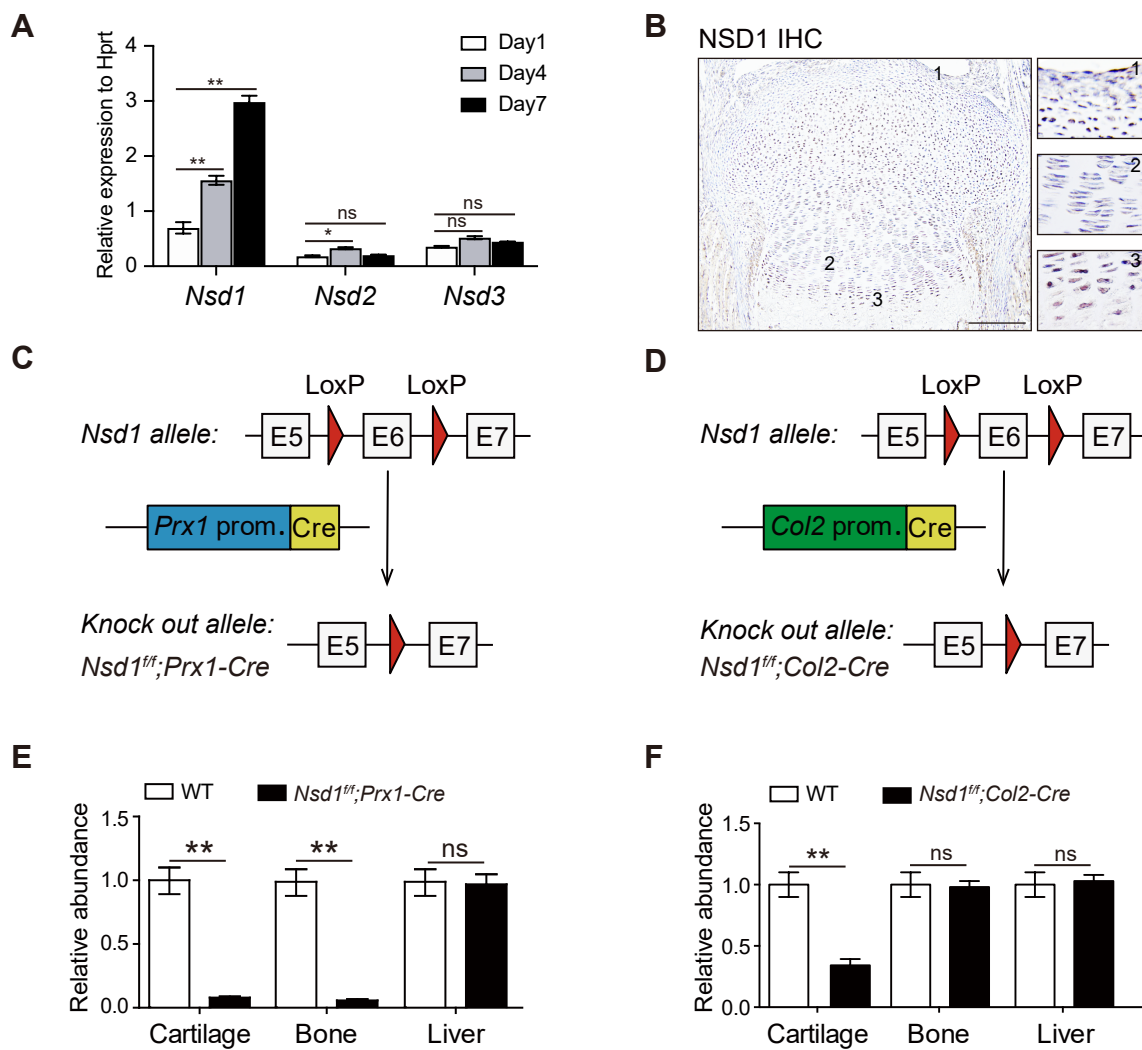

Fig. S2

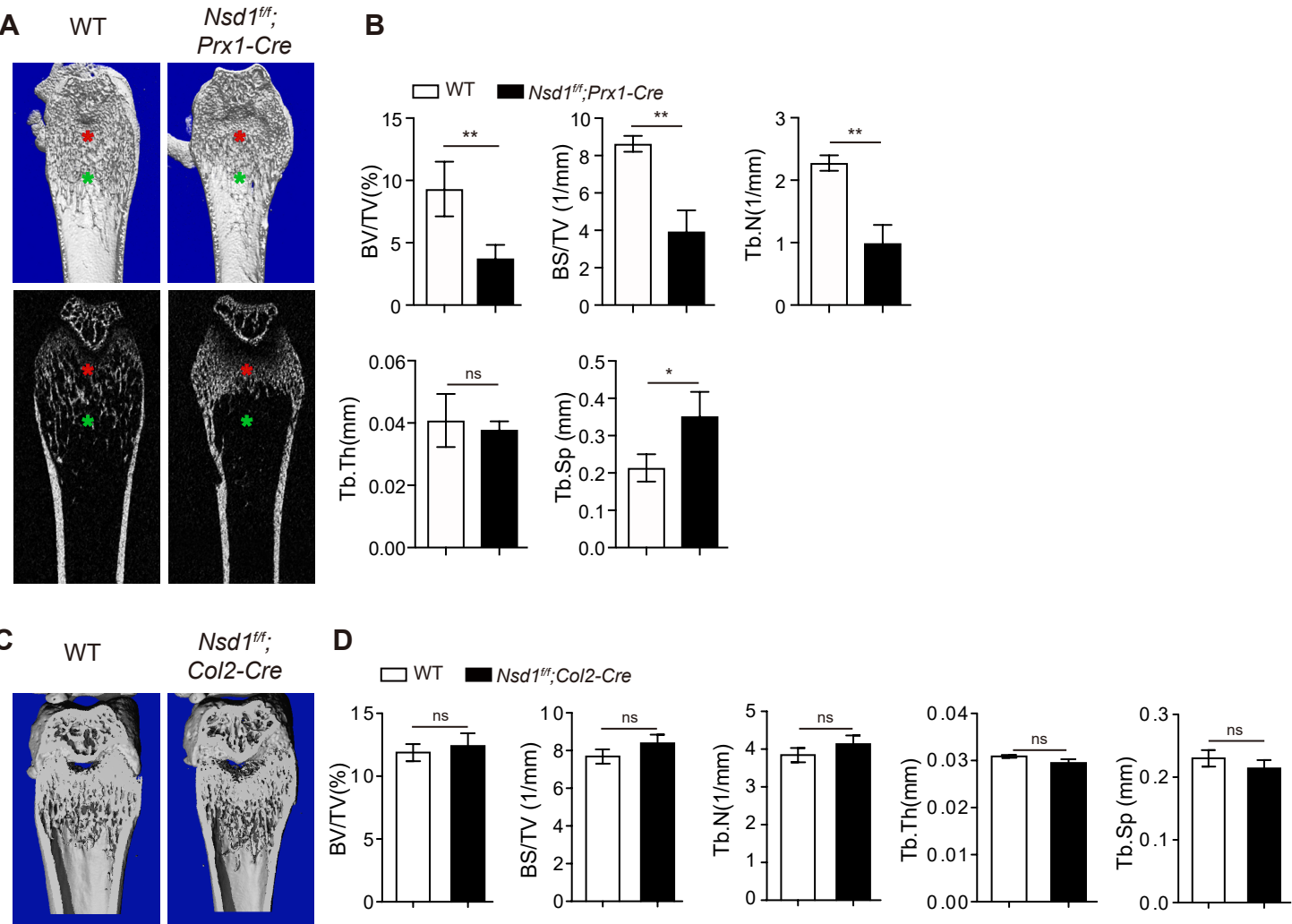

Fig. S3

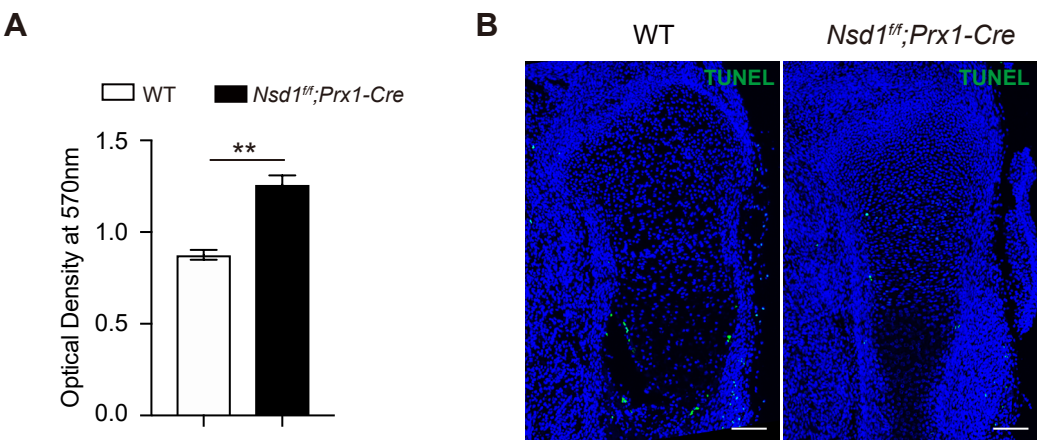

**Fig. S4**

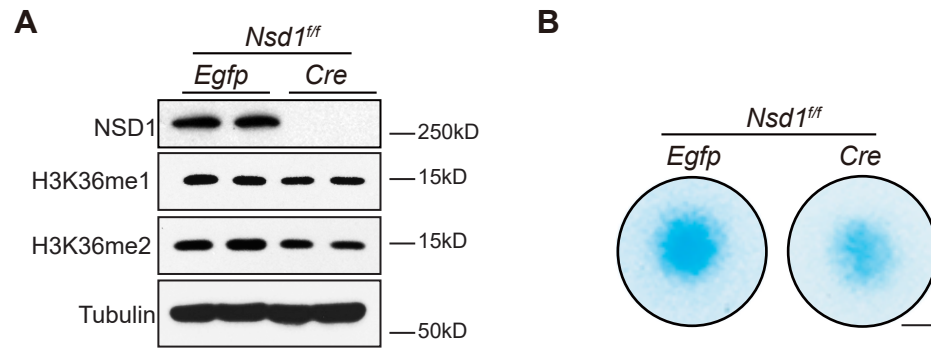

Fig. S5

A

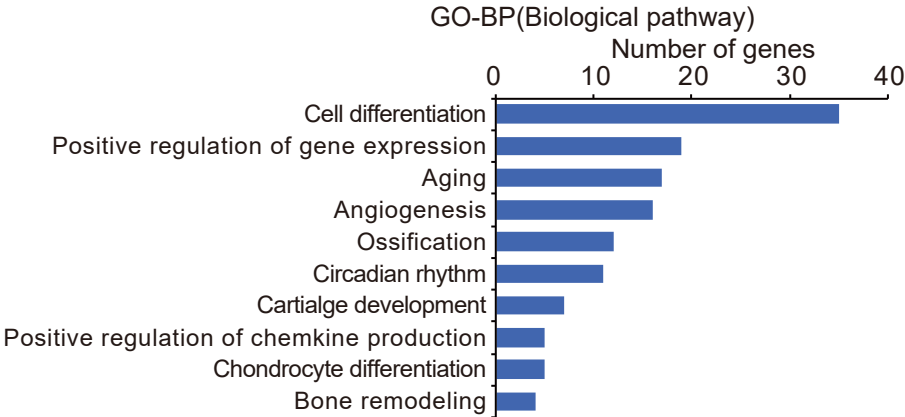

B

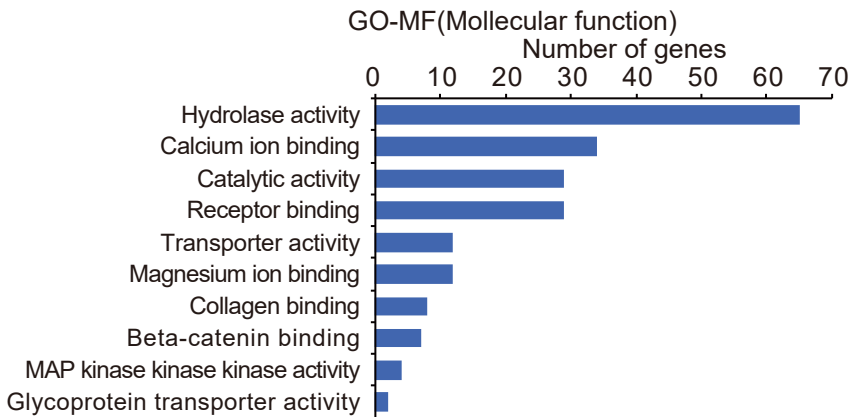

C

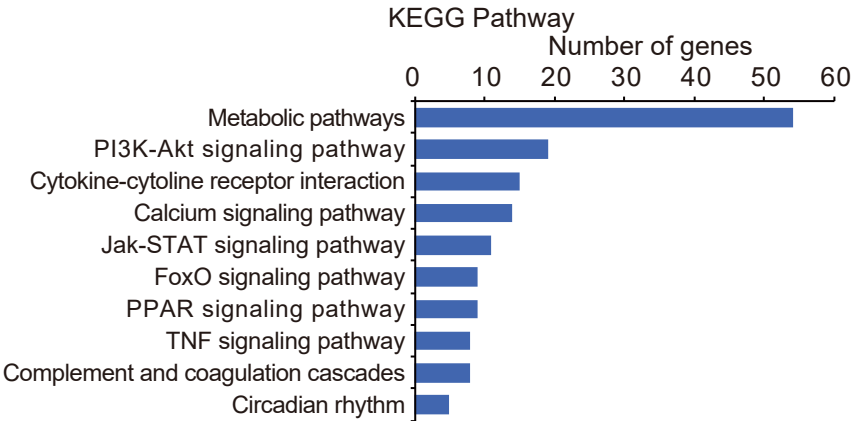

Fig. S6

A

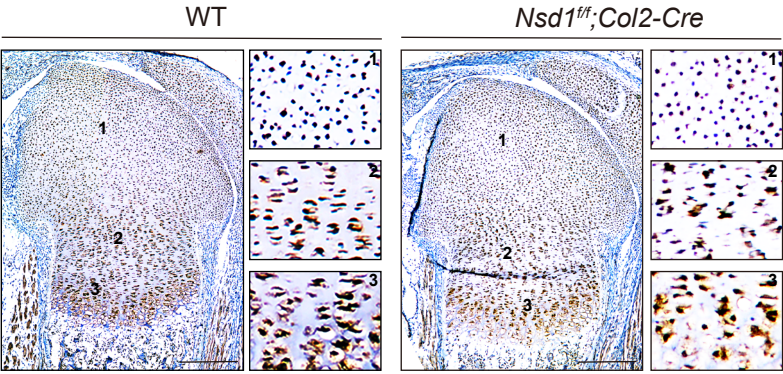

Fig. S7

A

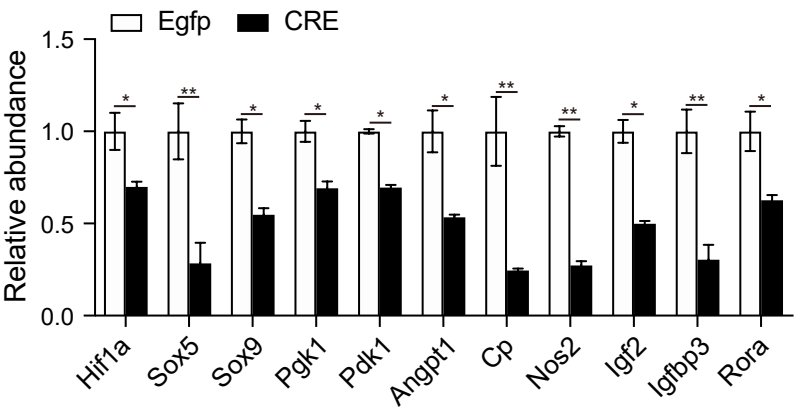

Fig. S8

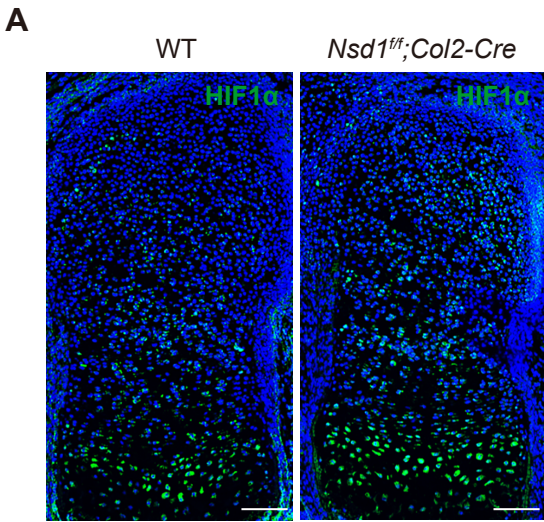

Fig. S9

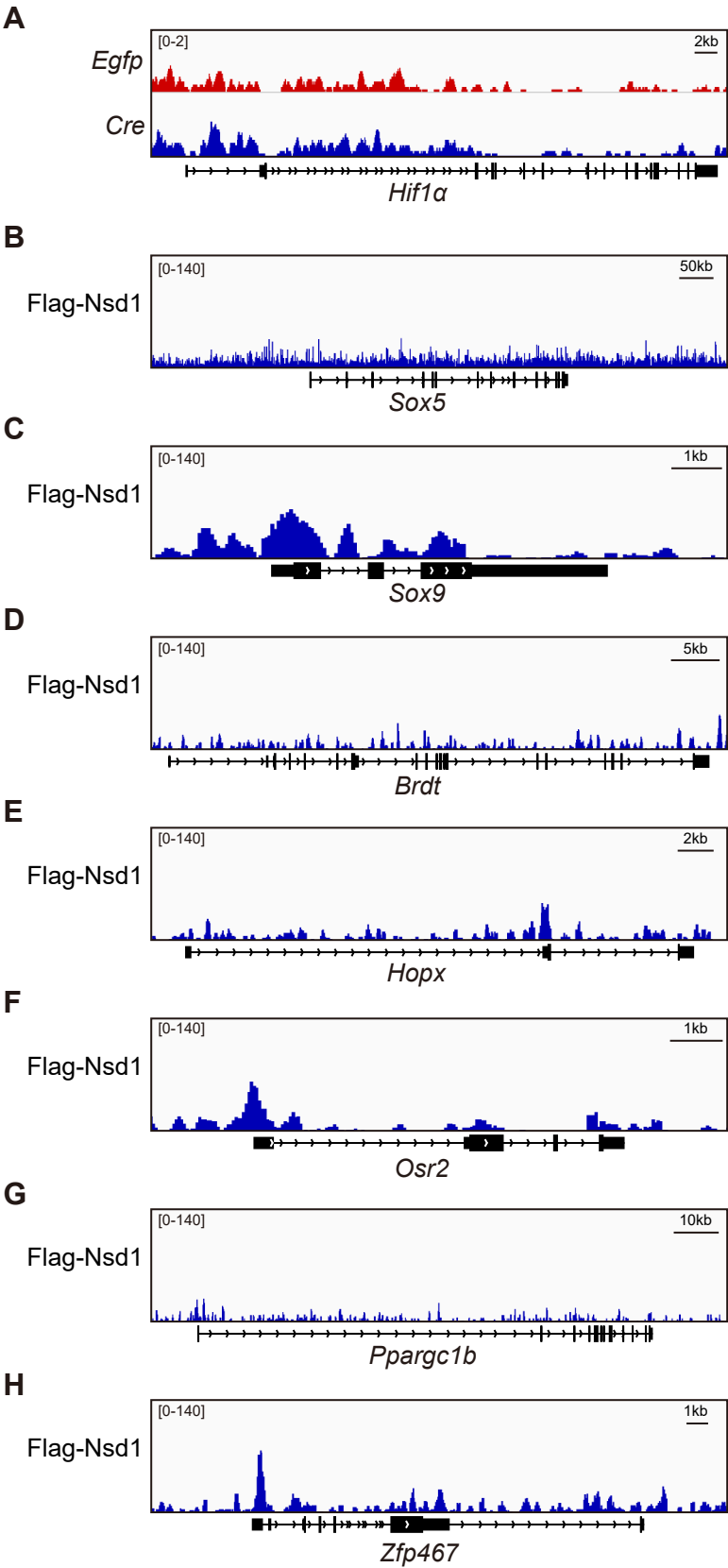

Fig. S10

A

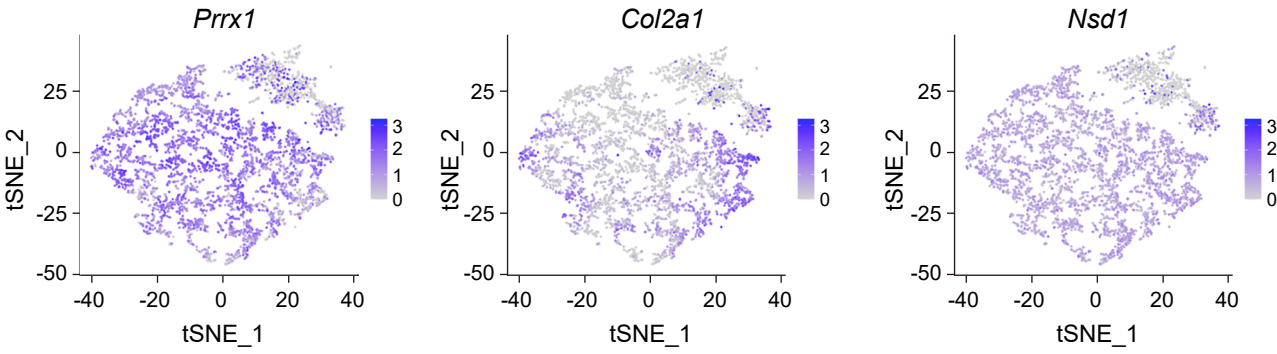

Supplement: Supplementary file 2 — Supplementary Information [file 41413_2021_148_MOESM2_ESM.pdf]
